# Structural and functional differentiation of bacterial communities in post-coal mining reclamation soils of South Africa: bioindicators of soil ecosystem restoration

**DOI:** 10.1038/s41598-020-58576-5

**Published:** 2020-02-04

**Authors:** Obinna T. Ezeokoli, Cornelius C. Bezuidenhout, Mark S. Maboeta, Damase P. Khasa, Rasheed A. Adeleke

**Affiliations:** 1 Unit for Environmental Sciences and Management, North-West University, Potchefstroom, 2520 South Africa; 2 Agricultural Research Council-Institute for Soil, Climate and Water, Arcadia, 0001 Pretoria, South Africa; 3 Institute for Integrative Systems Biology (IBIS), Universite Laval, Quebec City, QC G1V 0A6 Canada; 4 Centre for Forest Research, Faculty of Forestry, Geography and Geomatics, Universite Laval, Quebec City, QC G1V 0A6 Canada

**Keywords:** Microbial communities, Environmental microbiology

## Abstract

Soil microbial communities are suitable soil ecosystem health indicators due to their sensitivity to management practices and role in soil ecosystem processes. Presently, information on structural and functional differentiation of bacterial communities in post-coal mining reclamation soils of South Africa is sparse. Here, bacterial communities in three post-coal mining reclamation soils were investigated using community-level physiological profiling (CLPP), enzyme activities, and next-generation sequencing of 16S rRNA gene. Inferences were drawn in reference to adjacent unmined soils. CLPP-based species diversity and proportionality did not differ significantly (*P* > 0.05) whereas activities of β-glucosidase, urease and phosphatases were significantly (*P* < 0.05) influenced by site and soil history (reclaimed vs unmined). Bacterial communities were influenced (PERMANOVA, *P* < 0.05) by soil history and site differences, with several phylotypes differentially abundant between soils. Contrastingly, predicted functional capabilities of bacterial communities were not different (PERMANOVA, *P* > 0.05), suggesting redundancy in bacterial community functions between reclamation and unmined soils. Silt content, bulk density, pH, electrical conductivity, Na and Ca significantly influenced soil bacterial communities. Overall, results indicate that bacterial community structure reflects underlying differences between soil ecosystems, and suggest the restoration of bacterial diversity and functions over chronological age in reclamation soils.

## Introduction

The soil ecosystem supports numerous interactions between living and non-living matter. These interactions are vital to the soil’s ecological processes and key ecosystem services^1,2^. However, anthropogenic disturbances of the soil ecosystem through agriculture, mining and other land use activities, negatively affect these vital interactions^3,4^.

Although relevant post-mining reclamation guidelines exist in South Africa for restoring mined-out areas to acceptable conditions^5^, appropriate comprehensive soil quality monitoring tools for ensuring the success of post-mining reclamation efforts are still lacking. Such soil quality monitoring tools will help elucidate the adequacy of current reclamation practices and could provide insights into the potential restoration of ecological roles. At present, several above-ground monitoring indicators for soil quality, including vegetation cover, erodibility, and compaction levels have been proposed and utilised on post-coal mining reclamation sites^6^. However, these above ground indicators do not provide a comprehensive assessment of soil health given that the soil ecosystem is multidimensional with respect to its biological, physical and chemical components^7^. In recent years, the suitability of soil biota as soil health indicators has received much attention^7–9^. Notably, the use of soil microbial indicators has been proposed for soil quality monitoring because diversity and structure of microbial community are sensitive to natural or anthropogenic disturbances^7–10^. The suitability of microbes as bioindicators is supported by the direct relationship between the diversity of soil microbial communities and soil ecosystem function^2,11–13^ as well as the key roles of soil microorganisms in nutrient cycling, plant growth promotion, ecological succession and energy flow in soil ecological food webs^8,14–18^. For the foregoing reasons and to determine the potential impact of such disturbances on soil health processes and function, several studies have investigated soil microbial community composition and function in anthropogenically- and naturally-disturbed environments^8,17,19,20^.

Few studies^19–22^ have explored the microbial community functions and structure along a time gradient of post-coal mining reclamations within South Africa. Yet, very little is known about the microbial species diversity and functional community structure in post-mining reclamation soils of South Africa and how these communities differ from those of soils in unmined areas. Furthermore, compared to previous microbial community diversity studies, recent advances in sequencing technologies now make it feasible to unravel microbial communities of environments at a much deeper depth and coverage^23,24^. Also, these techniques provide a glimpse into potential ecological functions of microbial communities at a high-throughput scale^25^. An in-depth study of the microbial ecology of coal mining soil environments will help unravel species diversity of reclamation soils for bioprospection purposes, and more importantly, for identifying potential biomarker species for monitoring soil ecosystem recovery and health over several years since reclamation. At present, such in-depth microbial community studies on post-coal-mining reclamation sites are, however, sparse.

Hence, the present study investigated the diversity and potential functions of bacterial communities in post-coal-mining reclamation soils of three purposefully selected active coal mines located in the coal-rich Emalahleni Highveld, Mpumalanga province (29° 3′ 36′′S, 25° 52′ 12′′E), South Africa (See Supplementary Fig. S1). The mining sites are hereafter referred to as sites X, Y and Z. The study was designed to investigate the influence of site and history (reclaimed vs unmined) on soil bacterial communities. In site X, a recently (<1 yr.) reclaimed site was sampled (hereafter referred to as ReclX), while in site Y, a 1.5-year-old reclaimed site was sampled (hereafter referred to as ReclY). In site Z, a reclamation site of 18 yr. was sampled (hereafter referred to as ReclZ). In addition, soil samples were collected from non-mining areas adjacent to each coal mine. These samples served as reference (designated RefX, RefY and RefZ for sites X, Y and Z, respectively). The sites were selected based on the availability of reclamation sites within cooperative mining companies. Because it was not possible to obtain replicate sites with the same age of reclamation within sites, the experiment design did not include age as a main factor but rather as a random variable.

The hypothesis that the microbial community structure and function in reclamation soil are impaired compared to unmined soils (soil history effect) was investigated. To test this hypothesis, three coal mining sites located in the coal-rich Mpumalanga province of South Africa were selected for the study. The diversity and predicted function of the microbial community were investigated by utilising a combination of enzyme assays, carbon substrate utilisation pattern as well as next-generation sequencing (NGS) of the V3-V4 region of the bacterial 16S rRNA gene.

## Results

### Soil physicochemical properties

The texture of reclamation soils was largely characterised as sandy-clay-loam, while the reference soils were characterised as sandy-loam (Table 1). On the average, pH ranged from 4.41–5.86 in reclamation soils, and 4.52–7.22 in reference soils. Organic matter (OM) ranged from 3.18–3.84% in reclamation soils and from 4.20–9.07% in reference soils while cation exchange capacity (CEC) ranged from 3.88–5.84 cmol ( + ) kg^−1^ in reclamation soils, and from 4.46–11.83 cmol (+) kg^−1^ in reference soils (Table 1). Pair-wise comparisons revealed significantly (Tukey HSD, *P* < 0.05) higher bulk density (BD) in reclamation soils compared to respective unmined soils at all sites. Significant (Tukey HSD, *P* < 0.05) differences in pH were only observed between reclamation soil and reference soils in site Y. Differences in NO_3_^−^N, PO_4_^3−^P, K, Cl^−^, electrical conductivity (EC) and OM between reclamation and unmined soil were not significant (Tukey HSD, *P* > 0.05) in all sites (Tables 1 and S1), whereas, differences in Ca, Mg CEC, particle size were significant (Tukey HSD, *P* < 0.05) between reclamation soil and reference soils at one or more sites Generally, reclamation soils were more acidic, more compacted (inferred from BD measurement), lower in OM, CEC and EC compared to their respective unmined reference soils (Table 1).

**Table 1:** Selected physico-chemical properties of soil.

Properties	Site X		Site Y		Site Z	
	Recl	Ref	Recl	Ref.	Recl	Ref
pH	4.41 ± 0.07^a^	4.52 ± 0.79^a^	5.86 ± 0.25^b^	7.22 ± 0.22^a^	5.36 ± 0.35^a^	5.19 ± 0.74^a^
Moisture (%)	0.56 ± 0.10^a^	4.52 ± 6.29^a^	1.37 ± 0.29^b^	2.65 ± 0.01^a^	1.06 ± 0.23^a^	0.68 ± 0.08^b^
OM (%)	3.77 ± 1.31^a^	5.10 ± 1.91^a^	3.84 ± 0.77^a^	9.07 ± 3.85^a^	3.18 ± 0.47^a^	4.20 ± 1.11^a^
BD (g cm^−3^)	1.79 ± 0.06^a^	1.45 ± 0.10^b^	1.88 ± 0.03^a^	1.68 ± 0.09^b^	1.76 ± 0.18^a^	1.37 ± 0.19^b^
EC (mS m^−1^)	4.01 ± 0.63^a^	4.59 ± 1.89^a^	5.12 ± 1.52^a^	3.91 ± 0.63^a^	4.467 ± 2.96^a^	4.63 ± 2.47^a^
NO_3_^−^N (mg kg^−1^)	2.03 ± 0.47^a^	3.74 ± 6.26^a^	0.48 ± 0.24^a^	1.97 ± 1.40^a^	0.73 ± 0.14^a^	1.49 ± 2.07^a^
NO_2_^−^N (mg kg^−1^)	0.06 ± 0.02^a^	0.08 ± 0.11^a^	0.01 ± 0.02^b^	0.29 ± 0.16^a^	0.05 ± 0.03^a^	0.26 ± 0.57^a^
PO_4_^3−^P (mg kg^−1^)	0.16 ± 0.08^a^	0.10 ± 0.03^a^	0.02 ± 0.03^a^	0.09 ± 0.07^a^	0.05 ± 0.09^a^	0.18 ± 0.32^a^
CEC (cmol (+) kg^−1^)	5.84 ± 0.42^a^	7.11 ± 3.44^a^	5.42 ± 1.31^b^	11.83 ± 2.66^a^	3.88 ± 0.91^a^	4.46 ± 0.85^a^
Sand (%)	77.33 ± 1.15^a^	74.80 ± 6.42^a^	69.5 ± 1.91^b^	76.00 ± 2.00^a^	72.80 ± 5.02^a^	76.40 ± 1.67^a^
Silt (%)	2.67 ± 1.15^b^	9.20 ± 5.40^a^	6.5 ± 1.00^a^	8.00 ± 0.00^a^	5.20 ± 1.79^a^	5.20 ± 1.10^a^
Clay (%)	20.00 ± 2.00^a^	16.00 ± 1.41^b^	24.00 ± 1.68^a^	16.00 ± 2.00^b^	22.00 ± 3.74^a^	18.40 ± 2.19^a^

Values are mean ± SD (*N* ≥ 3). EC, Electrical conductivity; CEC, cation exchange capacity; OM, Organic matter; BD, Bulk density. Different superscript alphabet letters for each between pairs of reclamation and reference soils per site are significantly different based on the parametric independent sample t-test or the non-parametric Mann-Whitney U test.

### Community-level physiological profiles (CLPP) and Enzyme activities

Differences in CLPP-based soil microbial community diversity (Shannon-Wiener index, *H*′) and evenness (species proportionality) were not significantly different amongst treatments (Wald chi-square test, *P* > 0.05) (Table S2). However, the highest mean *H*′ was observed in site X, while the lowest *H*′ was observed in site Z. However, *H*′ increased with increasing age of reclamation, with greater *H*′ obtained in the older reclamation site (ReclZ) compared to the adjacent unmined soil (Table S2).

Soil β-glucosidase activity, urease activity, alkaline- and acid- phosphatases activities were significantly influenced (Wald chi-square test, *P* < 0.05) by cross-level interaction of the fixed factors (Table 2). Overall, lower activities of β-glucosidase activity, urease activity, alkaline- and acid- phosphatases were observed in reclamation soils compared to corresponding adjacent reference soils in each site (Table 2), with the most recently re-vegetated soil (ReclX) having the significantly (Wald chi-square test, *P* < 0.05) lowest soil enzyme activities among the reclaimed soils except for Alkaline-phosphatase activity (Table 2). In site Y, the average activities of β-glucosidase, acid phosphatase and urease were approximately four, fourteen and three times higher, respectively, in the reference soil compared to those of ReclY soil (Table 2). Furthermore, high intra-site variations (standard deviations) were observed in enzyme activities, especially for the phosphatases (Table 2). The trend in the mean values of all enzyme activities among reclamation soils was in the increasing order of ReclX < ReclY < ReclZ (Table 2), suggesting that enzyme activities are directly related to the age of reclamation sites.

**Table 2:** Soil enzyme activities.

Site	Soil group (Sample size)	ß-glucosidase (P-nitrophenol µg/g/h)	Alk-phosphatase (P-nitrophenol µg/g/h)	Acid-phosphatase (P-nitrophenol µg/g/h)	Urease (NH_4_−N µg/g/2 h)
Site X	ReclX (N = 3)	66.18 ± 18.86^c^	148.59 ± 54.60^b^	869.95 ± 153.65^c^	5.02 ± 1.07^c^
	RefX (N = 5)	457.14 ± 64.42^ab^	182.87 ± 39.09^b^	1603.13 ± 48.97^a^	21.25 ± 7.44^abc^
Site Y	ReclY (N = 5)	175.66 ± 74.66^bc^	155.17 ± 73.16^b^	1186.49 ± 233.67^bc^	16.44 ± 5.56^bc^
	RefY (N = 3)	693.83 ± 130.37^a^	2142.69 ± 633.53^a^	1437.06 ± 378.83^ab^	45.42 ± 25.38^a^
Site Z	ReclZ (N = 5)	529.79 ± 113.28^a^	226.16 ± 110.03^b^	1640.53 ± 102.21^a^	28.95 ± 7.26^ab^
	RefZ (N = 5)	494.92 ± 270.51^ab^	256.77 ± 142.08^b^	1536.95 ± 170.33^ab^	27.42 ± 12.36^abc^

Values (mean ± SD) followed by different superscript letters across columns are significantly different (Wald chi-square test, *P* < 0.05) based on interactions between fixed factors “site” and “history” in the mixed linear model. Alk, Alkaline.

### Diversity and community structure of soil bacterial operational taxonomic units (OTUs)

**Figure 1:**
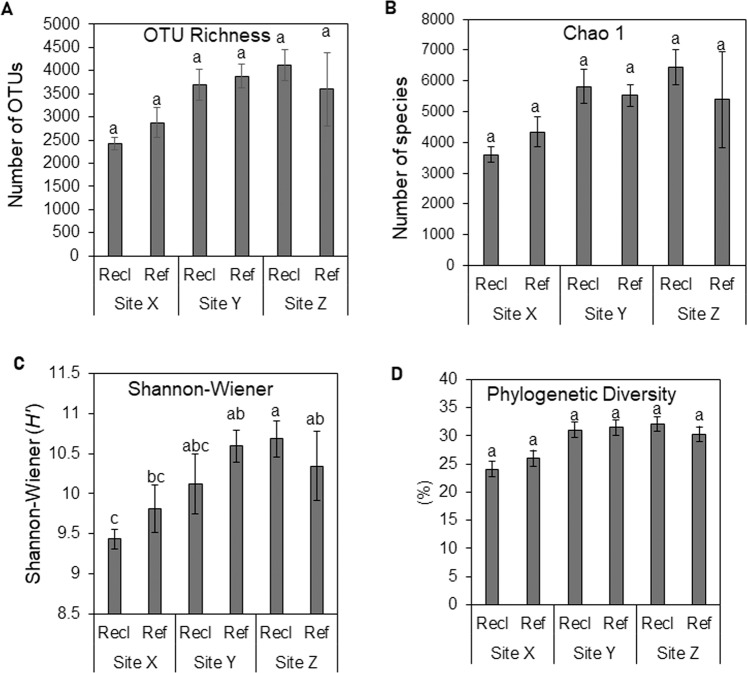
16S rRNA-based OTU diversity indices. (**A**) OTU richness. (**B**) Chao 1 richness estimate. (**C**) Shannon-Wiener index of diversity. (**D**) Phylogenetic diversity based on PD whole tree. Values with different superscript letters are significantly different (Wald chi-square test, *P* < 0.05) based on the interaction effect in a mixed linear model. The main effect “site” is significant (Wald chi-square test, *P* < 0.05) for OTU richness, Chao1 and phylogenetic diversity. Differences between reference and reclamation area (pair-wise comparison) for each site is not significant (Mann-Whitney U, Test, *P* < 0.05).

After rarefaction of partial 16S rRNA gene sequences to even depth of 19 500 (see rarefaction curve in Fig. S3), the number of OTUs (97% 16S rRNA gene sequence similarity) common to site-pairs of reference and reclamation soils was highest in site Z (Fig. S4). Lowest OTU richness and diversity were observed in the most recently reclaimed ReclX soil, whereas ReclZ had the highest species richness and diversity in comparison to other reclamation sites (Fig. 1). Based on a mixed linear model, differences in OTU richness (Fig. 1A), Chao1 richness estimation (Fig. 1B) and phylogenetic diversity (Fig. 1D) are only significant (Wald chi-square test, *P* < 0.05) between sites (main effect of “site” averaged over soil history), with OTU richness and phylogenetic diversity values of site X significantly lower (Wald chi-square test, *P* < 0.05) than those of other sites (Fig. 1A,D). The interactions between fixed factors for Shannon-Wiener index of diversity is significant (Wald chi-square test, *P* < 0.05) (Fig. 1C). However, pair-wise comparisons of these alpha-diversity indices between reclamation and reference soils for each site were not significant (Mann-Whitney U test, *P* > 0.05).

**Figure 2:**
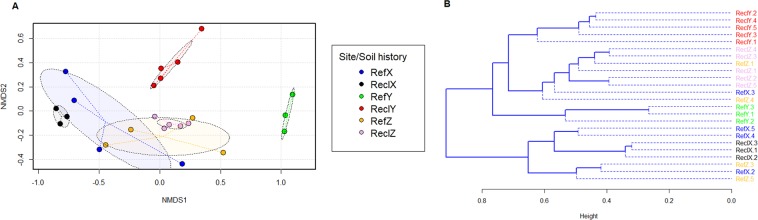
Bray-Curtis dissimilarity between bacterial communities (97% 16S rRNA gene similarity). (**A**) Non-metric dimensional scaling (nMDS) plot. (**B**). UPGMA hierarchical cluster dendrogram. Dotted lines in the nMDS plot show the distance of every sample to its group centroids in multivariate space, while ellipses show 95% confidence intervals (standard error) in multivariate space around group centroids. The stress of the nMDS plot is 0.067 (also see Fig. S5). Differences in multivariate space are significant for site and history interactions (PERMANOVA R^2^ = 14.8%, *P* = 0.001). See Table S3 for pair-wise PERMANOVA test for reference and reclamation soils per site. nMDS and UPGMA cluster dendrograms were constructed by using the vegan (v. 2.5.5) and dendextend (v. 1.12.0) packages of R software (https://cran.r-project.org/), respectively.

In multivariate space, differences between bacterial community structure (97% 16S rRNA gene similarity OTUs) were differentiated between reclamation (ReclY) and unmined soil (RefY) in site Y, but less differentiated in site X and site Z (Fig. 2). Between reclamation soils, the bacterial community structure of ReclY and ReclZ were closely similar but jointly less similar to those of ReclX (Fig. 2A). The community differentiation pattern observed in multivariate space (Fig. 2A) is also supported by the hierarchical cluster dendrogram shown in Fig. 2b. For the whole bacterial community dataset, permutational analyses of variance (PERMANOVA) revealed that the interactions between “site” and “soil history” effects (R^2^ = 14.8%, *P* = 0.001) are significant. Whereas for the pair-wise comparisons, only differences between ReclY and RefX are significant (PERMANOVA R^2^ = 51.5%, *P* < 0.026; PERMDISP *P* = 0.111) (Fig. 2 and Table S3). Overall, these trends in bacterial community structure suggest the influence of chronological age-related factors in driving differences among reclamation areas as well as the influence of other confounding factors that may be specific to each site or sampled area.

### Dominant and differentially abundant phylotypes between reference and reclamation soils

**Figure 3:**
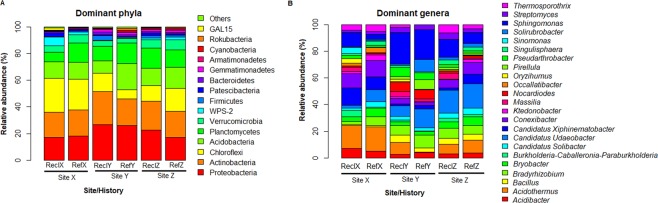
The average relative abundance of dominant (>1%) phylotypes per site. (**A**) Phylum. (**B**) Genus. Phylotypes with average relative abundance <1% and those which were unclassified at genus taxa level are excluded from the plot. Bar plots were constructed based on average relative abundance per group.

Taxonomically, the dominant (≥1% relative abundance on the average) classifiable OTUs belonged to 14 phyla and 24 genera (Fig. 3). Proteobacteria, Actinobacteria, Chloroflexi, Acidobacteria, Planctomyces, Verrucomicrobia and candidate phylum division WPS-2 were among the most relatively abundant phyla across sample groupings. The relative abundance of Firmicutes exceeded 1% only in soils from site Z (Fig. 3A). A large proportion (30–48%) of 16S rRNA gene sequence were unclassified at the genus taxonomic rank (data not shown). Of the classifiable phylotypes, the genera *Acidibacter*, *Acidothermus*, *Bacillus*, *Bradyrhizobium*, *Burkholderia-Caballeronia-Paraburkholderia*, *Candidatus Udaeobacter*, *Candidatus Xiphinematobacter*, *Conexibacter* and *Sphingomonas* were relatively abundant across soils (Fig. 3B). *Acidothermus*, *Sphingomonas* and *Candidatus Udaeobacter* were relatively most abundant in both soil types for sites X, Y and Z, respectively, (Fig. 3B).

**Figure 4:**
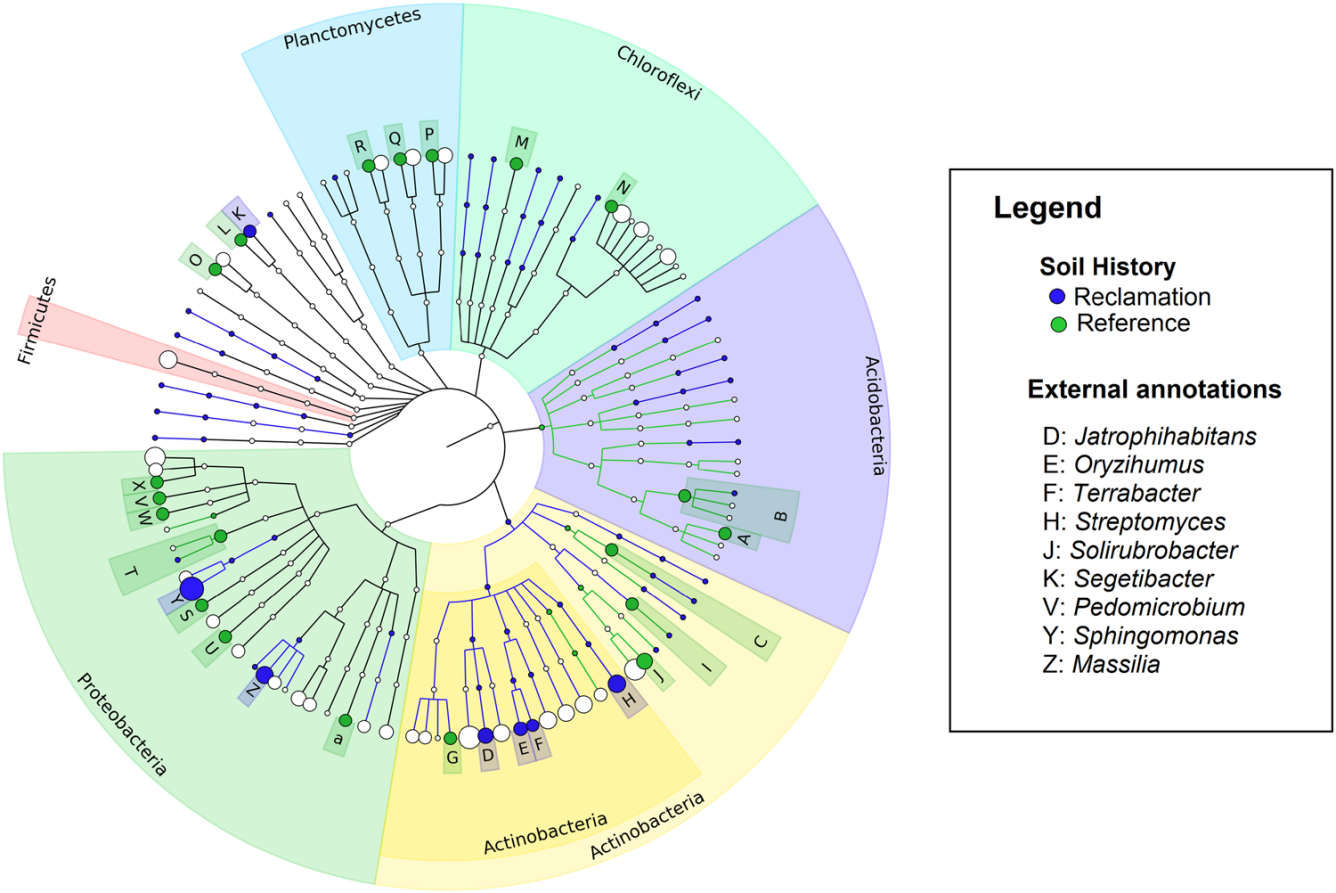
Cladistical representation of the top 100 differentially abundant (LDA > 2.0, Mann-Whitney U test, *P* < 0.05) features amongst soil bacterial communities. The features are ordered based on relative abundance. Rings (from inner to outer rings) 1, 2, 3, 4, and 5 represent phylum, class, order, family and genus taxonomic ranks. Only annotations for discriminant features classifiable at the genus taxonomic rank (ring 5) are shown in the legend. See supplementary Table S4 for the FDR-adjusted *P*-values for the genus-level discriminative features. Cladogram was constructed using GraPhlAn software (v. 0.9.9)^89^.

A total of 194 discriminative features (Mann-Whitney U test, *P* < 0.05, Linear discriminant analysis (LDA) score > 2) were identified between reclamation and reference soils following a sample-wide LDA Effect size (LEfSe) analysis (data not shown). The top 100 discriminative features are shown in Fig. 4. Of these, the classifiable genus-level features differentially more abundant in reclamation soil compared to references soil include *Jatrophihabitans*, *Massilia*, *Oryzihumus*, *Segetibacter*, *Sphingomonas, Streptomyces* and *Terrabacter* (Fig. 4 and Table S4). Whereas, *Solirubrobacter* and *Pedomicrobium* were differentially more abundant in reference soil compared to reclamation soils (Fig. 4 and Table S4).

**Figure 5:**
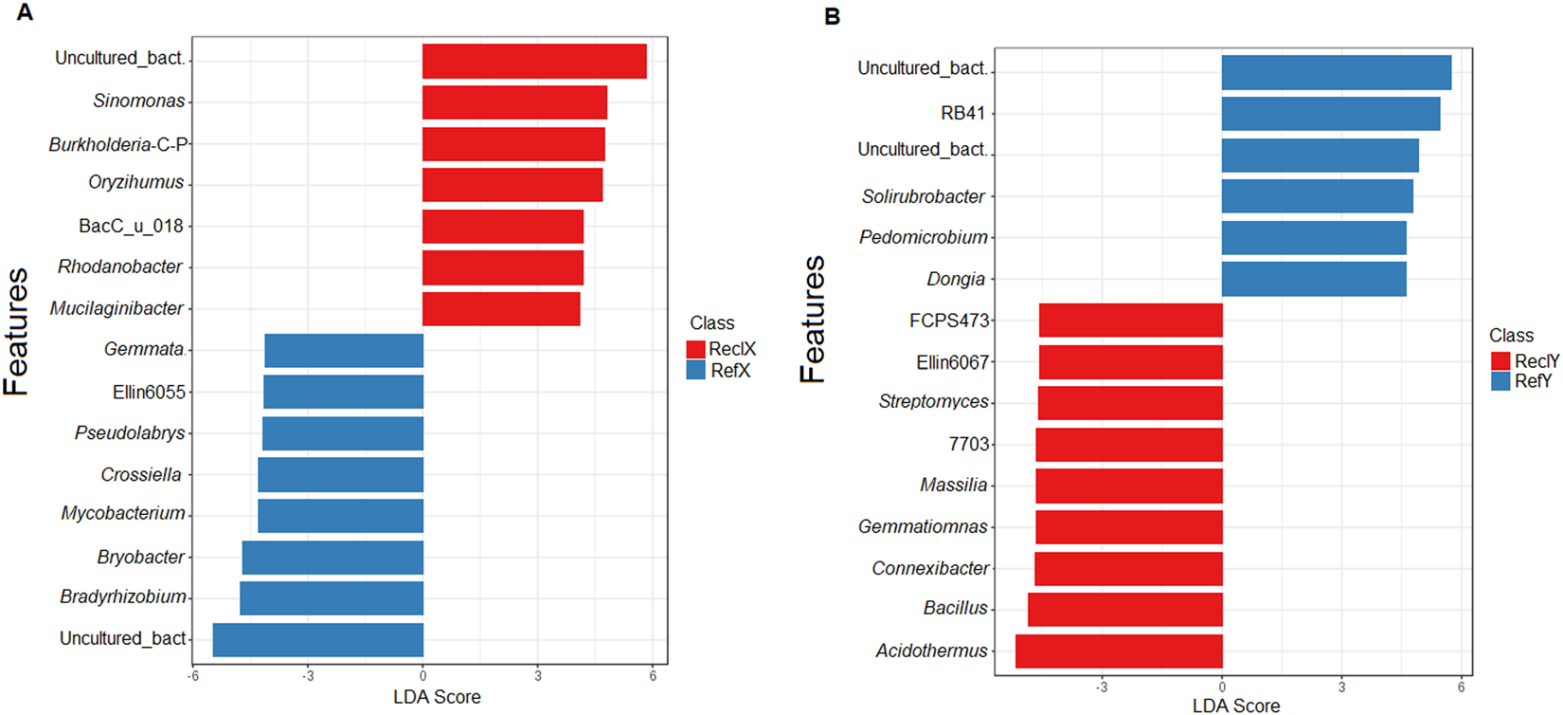
Differentially abundant genera between reclamation and reference soils (**A**) Top 15 discriminant (LDA score > 2.0, FDR-adjusted *P*-value < 0.1) genera in Site X. (**B**) Top 15 discriminant (LDA score > 2.0, FDR-adjusted *P*-value < 0.3) genera in Site Y. Differential abundance and bar plots were determined and generated, respectively using Linear Discriminant Analysis (LDA) Effect size (LEfSe) via the web-based Microbiome Analyst tool (www.microbiomeanalyst.ca). Burkholderia-C-P, *Burkholderia*-*Caballeronia*-*Paraburkholderia*; Uncultured_bact., Uncultured bacteria.

In the context of this study, phyla Planctomycetes and Candidate phylum WPS-2 were discriminant (false discovery rate (FDR)-adjusted *Q* < 0.3, LDA score > 2) between reference and reclamation soils in both sites X and Y (Fig. S6A and S6B), with Planctomycetes more abundant in reference soils and Candidate phylum WPS-2 more abundant in reclamation soils at both sites X and Y. The top 15 (ranked by *P*- values) differentially abundant (FDR-adjusted *Q* < 0.1 or 0.3, LDA score > 2) features at the genus taxonomic rank for site X and Y are shown in Fig. 5. In site X, *Sinomonas*, *Burkholderia-Caballeronia-Paraburkholderia*, *Oryzihumus*, *Rhodanobacter* and *Mucilaginibacter* were differentially (FDR-adjusted *Q* < 0.1, LDA score > 2) more abundant in ReclX compared to RefX, whereas *Bradyrhizobium*, *Bryobacter*, *Mycobacterium*, *Crossiella*, *Pseudolabrys*, Ellin6055 (Family: *Sphingomonadaceae*) and *Gemmata* were differentially more abundant in RefX compared to ReclX (Fig. 5A). In site Y, RB41 (Family: *Pyrinomonadaceae*), *Solirubacter*, *Pedomicrobium* and *Dongia* were differentially more abundant in ReclY compared to RefY, whereas, *Acidothermus*, *Bacillus*, *Conexibacter*, *Gemmatimonas*, *Massilia*, 7703 (Family: *Ktedonobacteraceae*), *Streptomyces*, Ellin6067 (unclassified Betaproteobacteria) and FCPS473 (Family: *Ktedonobacteraceae*) were differentially more abundant in RefY compared to ReclY (Fig. 5B). Unlike site X and Y, no differentially abundant *P-* > 0.05, LDA score > 2) phylotypes were observed between reclamation (ReclZ) and reference (RefZ) soils in site Z (data not shown).

### Predicted functional diversity and differentially abundant nutrient-cycling KO terms

**Figure 6:**
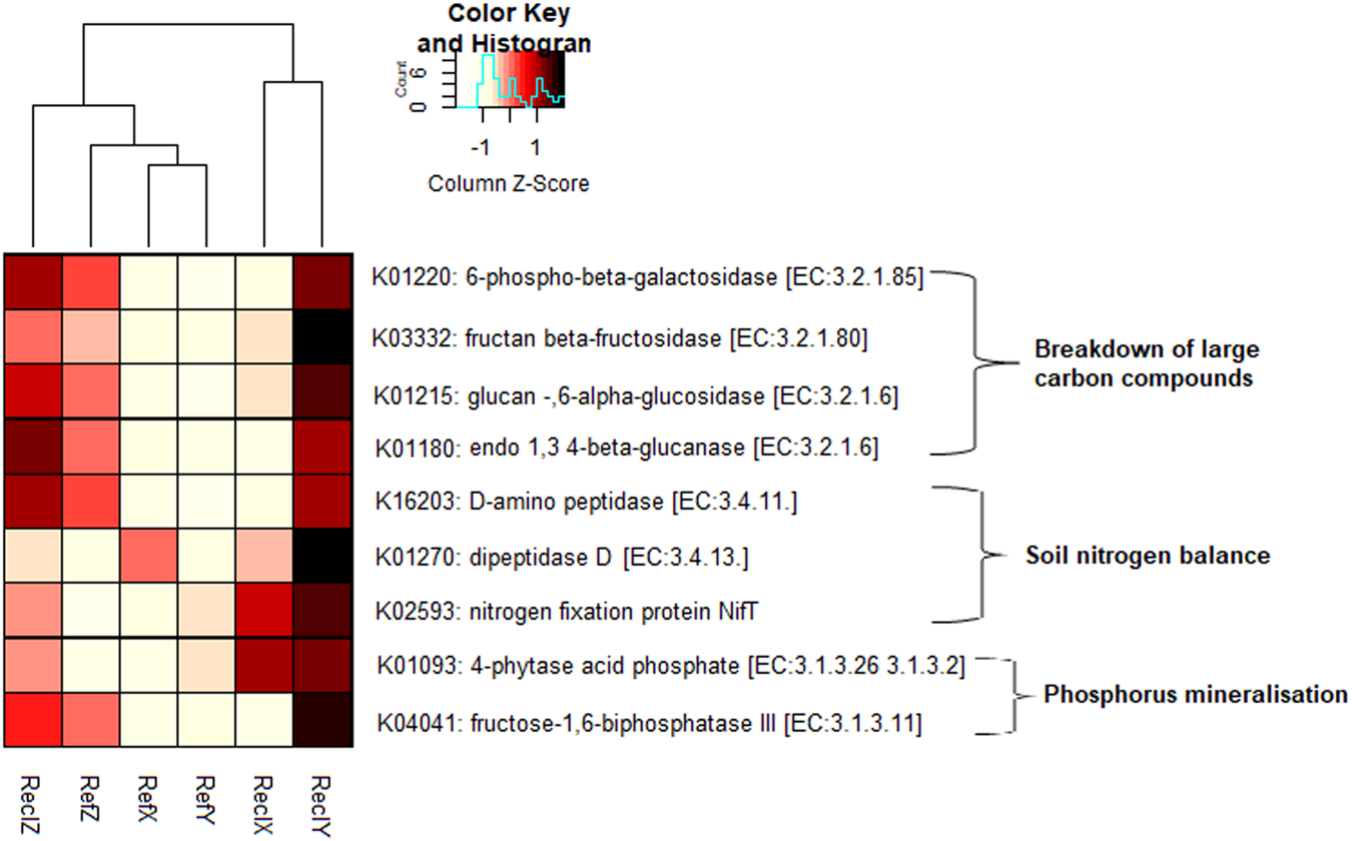
Relative abundance of differentially abundant (Mann-Whitney U test FDR-adjusted *P* < 0.1, indicator value > 0.6) KEGG Orthology terms related to carbon, nitrogen and phosphorus. Hierarchical cluster dendrogram are based on the average Bray-Curtis distances between soil groups. Relative abundance (colour key) is scaled across rows. KOs were generated from normalised 16S rRNA copy numbers and a set of pre-computed metabolic reference profiles based on the Kyoto Encyclopaedia of Genes and Genomes (KEGG) database (http://www.genome.jp/kegg/).

The average fraction of OTUs which mapped onto the Kyoto Encyclopaedia for genes and genomes (KEGG) organisms was not significantly different across soils (site and history) (Table S5), suggesting that any comparisons in the diversity of KEGG orthology (KO) terms between soil groups are valid. A total of 6448 KO terms were predicted from all OTUs. The predicted KO diversity (or functional) profile of soil bacterial communities significantly differed between sites (PERMANOVA R^2^ = 40.9%, *P* = 0.001; PERMDISP *P* = 0.78). However, unlike the bacterial genetic diversity, the predicted community functional diversity was less differentiated (Fig. S7) and was not significantly influenced by soil history (PERMANOVA R^2^ = 7.91%, *P* = 0.045; PERMDISP *P* = 0.016) and interactions between site and history (PERMANOVA R^2^ = 9.74%, *P* = 0.112). From a subset of KO terms/enzyme involved in the metabolism of carbon, phosphorus and amino acid/nitrogen-containing compounds, nine differentially abundant (FDR-adjusted *P* < 0.1, Indicator value > 0.6) KO terms between reclamation and reference soils were identified (Fig. 6). Most of the differentially abundant predicted KO terms, including fructan beta-fructosidase [EC:3.2.1.80], dipeptidase [EC:3.4.13], fructose-1,6-biphosphastase III [EC:3.1.3.11] were highest in ReclY. Among reclamation soils, the youngest reclamation area (ReclX) had the least predicted abundance of the differentially abundant KOs (Fig. 6). However, based on Bray-Curtis distances, close associations (as observed from the hierarchical cluster dendrogram) were observed between ReclX and ReclY compared to ReclZ (Fig. 6). In addition, the oldest reclamation area (ReclZ) was similar to the adjacent reference area and other reference sites (Fig. 6) thereby suggesting that bacterial community functional restoration (similarity to unmined reference) are a function of chronological age since reclamation.

### Relationship between soil physicochemical properties and biological properties

Correlations between carbon utilisation-based microbial diversity indices and soil-physicochemical properties were not significant (*P* > 0.05) (Table S6). However, positive correlations were observed between beta-glucosidase activity and Na content (Spearman rank correlation coefficient *r* = 0.55, *P* = 0.001) and between beta-glucosidase activity and Ca content (*r* = 0.413, *P* = 0.045) (Table S6). In contrast, a significant negative correlation was observed between NO_3_^−^ and soil urease (*r* = −0.45, *P* = 0.027. All other correlations between soil biological parameters and physicochemical properties were not significant (*P* > 0.05) correlations (Table S6).

**Figure 7:**
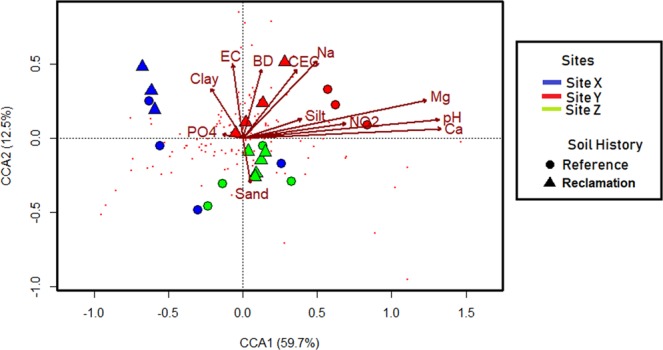
Canonical correspondence analysis triplot depicting the relationship between the relative abundance of OTUs (genus taxa rank) and constraining variables. The first species axis is significant (*P* < 0.05). Tiny red dots indicate genera. Some constraining variables showing collinearity with one or more variables are excluded from the final model plot.

The CCA model for the triplot depicted in Fig. 7 is significant (ANOVA F = 1.61, *P* = 0.004). Soil physicochemical properties explain up to 94.3% of the total variation in the bacterial community composition across sites. Site-specific clustering, as opposed to soil history-based clustering, was observed, particularly in site Z (Fig. 7). Of the soil physicochemical variables included in the CCA model, only silt content, BD, pH, EC, Na and Ca were significant (Table S7). Overall, variations in the microbial community structure in site Y were largely influenced by differences in soil physicochemical properties, while sites X and Z were least influenced.

## Discussion

Soil bacterial communities contribute to soil ecosystem processes by regulating the decomposition of organic matter and mineralisation of soil nutrients, amongst others. Such functions of soil microorganisms are vital for soil ecosystem functioning and health. The present study investigated the soil bacterial community structure and functions in post-coal mining reclamation and unmined reference areas across three coal mining sites. Furthermore, a differential abundance testing was performed to identify species and predicted functions which are discriminatory between these two areas towards identifying potential bioindicator species or functions for monitoring soil ecosystem health in mining-disturbed soil environments.

The textural classification of reference soils differed from reclamation soils at all sites. The lower silt fraction and organic matter content of reclamation soils suggest that reclamation soils were less fertile compared to the reference sites. This is because the silt and organic matter content of the soil are vital to nutrient retention and availability as well as for improved soil aeration and structure^26^. Furthermore, the higher clay content of reclamation soils may predispose high compaction, poor aeration and poor penetration of plant roots. Reclamation soils generally had higher bulk density compared to unmined soils (Table 1). These observations of higher bulk density in disturbed soils can be attributed to compaction from the use of heavy machinery during the replacement of soils in mined-out areas, and the higher fraction of clayey fractions in reclamation soils and they are similar to observations made in earlier studies on stockpiles and reclamation areas^3,6,27^. Although differences in pH were not significant between soil histories, pH values in most soils were sub-optimal (acidic) for most crops^28^. The low pH (acidity) values are likely due to the leaching of basic ions or oxidation of soil sulphide compounds^6,29^. The observation of no significant differences in most chemical properties (e.g. pH, EC, CEC, OM) (Table 1) between reclamation sites and references sites are similar to the earlier observations by Paterson *et al*.^6^, Claassens *et al*.^21^ and Claassens *et al*.^19^, who observed no major differences in selected chemical properties between post-coal mining reclamation sites and unmined adjacent reference soils in South Africa. These observations can be attributed to the fact that the reference soils are not being utilised, and hence are not replenished with nutrients and organic matter. From a land-use perspective, soils in both reclamation and reference soils will require liming and fertilisation prior to use in agriculture or pasture^26,28^.

The observations of no differences in the Shannon-Wiener index of diversity and evenness derived from community-level physiological profiling (CLPP) (Table S1) suggests that the microbial communities of reclamation soils and reference soils have similar metabolic capabilities for utilising the diverse carbon sources provided in the assay. The evenness values also suggest that the proportion of the physiologically distinct (with respect to carbon utilisation) species in the community are similar across soils. In a study by Markowicz *et al*.^17^, no differences were observed in the functional diversity (as determined by carbon utilisation profiles) of plant-associated microbial communities in coal mining soil stockpiles - of different ages of natural reclamation. Summarily, these observations hint at functional redundancy within the microbial communities for metabolising carbon. However, the observation of higher microbial diversities (*H*′ index) in the reference soil compared to reclaimed soils in most of the sites (Table 2) are similar to observations of Lewis *et al*.^30^ who observed that based on CLPP, higher microbial diversity was observed in unmined soils compared to those in post-bauxite reclamation soils irrespective of reclamation age. The reduction in Shannon-Wiener index with increasing age of reclamation soils may be due to the development of a streamlined functional community in the older reclamation soils^18,31,32^.

The average β-glucosidase, urease, alkaline- and acid phosphatases activities were lower in most reclamation soils compared to the adjacent reference soils, suggesting potentially lower (compared to reference soils) availability of carbon (e.g. cellobiose), N-mineralization (ammonification) and phosphates in most reclamation soils^33–35^. These enzymes are utilised as soil quality indicators for monitoring the response of soil organisms to disturbances in different soil ecosystems^9,35–38^. Furthermore, the significant interaction effect of fixed factors on these enzyme activities suggests that they are sensitive to differences between sites and soil disturbances^9,37,39^. The values obtained for enzyme activities in all reclamation soils are within the range obtained for post-oil mining reclamation soils^40^ and post-coal mining reclamations soils^19,41^. The comparability of the enzyme activity values to those obtained fertilised soils under cultivation^42,43^ and pasture soils^37^ suggest similar ecosystem functional capabilities of the reclamation soil microbial communities, and thus, provide indications of the potential suitability of these soils for agriculturally-related uses. The overlaps in the range of enzyme activities values between reclamation soils and reference soils observed in most sites are similar to the reports of Claassens *et al*.^19^ and Claassens *et al*.^41^ on post-coal mining chronosequences. The increase in the activities of β-glucosidase, urease, alkaline- and acid phosphatases with increasing age of reclamation soils suggests the restoration of ecological functions of soil microbial communities over chronological age. Importantly, the activities of these soil enzymes are usually related to the soil physicochemical properties, particularly soil carbon, phosphate and nitrogen content^44^. Positive correlations between beta-glucosidase and organic matter were observed, although not significant, while the correlations between alkaline- or acid -phosphatases and phosphate content of the soil were very weak. These observations are likely due to the specificity of β-glucosidase to cellobiose, which is only a fraction of the total organic matter, and because underlying effects of site, soil histories or both factors on soil organic matter and phosphates were not considered in the correlational analysis.

Microbial community richness and diversity is linked to the plasticity of soil ecosystem functionality^12,13^. The highest number of OTUs shared among pairs of reclamation and reference soils were between the oldest reclamation soil (ReclZ) and the adjacent reference suggesting that the older reclamation soils were most similar to the reference soils in terms of bacterial species richness. This information, along with the generally higher species richness and diversity in older reclamation areas (ReclY and ReclZ) compared to the more recently reclaimed area (ReclX), suggests the restoration of pre-mining disturbance bacterial community and function over chronological age. These observations agree with the results obtained for enzyme activity assays discussed earlier. Similarly, high-throughput sequencing-based studies by Li *et al*.^45^ and Hou *et al*.^46^ on the microbial diversity and community structure in post-coal mining sites in China indicated lowest bacterial diversity in more recent reclamation soils compared to older reclamation soils. Furthermore, microbial community diversity and structure across post-mining reclamation sites indicated restoration of microbial communities in older reclamation sites when compared to unmined soils^45–47^. The within- and between- dissimilarity in bacterial communities of the reference sites may be due to other (other than mining) unknown anthropogenic or site-specific influences such as roads and grazing since the mining sites occupy a very large area and are close to urban areas^19^. The significant *P*-values obtained for the fixed-factor PERMANOVA analyses suggest that, indeed, the bacterial community composition and structure are influenced by soil history/disturbances and may vary from one site to another consequent on the prevailing factors at each site. Such observations are expected given that the movement of soil and entire reclamation practice may vary slightly from one mining company to the next. Previous findings have also shown that bacterial communities are differentiated across landscapes^48^, and are sensitive to the prevailing soil management practice^8,10^.

For the first time, we report the bacterial community diversity of post-coal mining reclamation soils in South Africa using high-throughput next-generation sequencing (NGS) technologies. Similar to the study of Li *et al*.^45^ on post-coal reclamation and unmined soils in China, several dominant and rare bacterial phylotypes, including Proteobacteria, Actinobacteria, Chloroflexi, Acidobacteria, Planctomyces and Verrucomicrobia were observed across reclamation and reference soils. Some species of these phyla contribute to the diverse microbial functions and process in the soil ecosystems, including processes that are critical to soil ecosystem sustainability^17,48,49^. The relatively abundant phyla included underexplored phyla divisions such as WPS-2. Until recent advances in NGS technologies, knowledge of the ubiquity, biogeography and potential roles of WPS-2 in the soil environment have been limited^49^. For example, based on metagenomic analyses, species in the candidate phylum WPS-2 are consistently associated with soil environments globally and possess capabilities (genes) for anoxygenic photosynthesis which is important towards sequestration of atmospheric carbon and the generation of biomass in the soil ecosystem^50,51^.

Similarly, the phyla Gemmatimonadetes and Nitrospirae remain underexplored with only a few isolates so far characterised. Some characterised species in the phylum Gemmatimonadetes are capable of carbon fixation^52^, while some species in the phylum Nitrospirae are involved in the biogeochemical cycling of soil sulphur, iron and nitrogen^53,54^. Nevertheless, a high proportion of unclassified taxa (not shown) indicate that the bacterial community of coal-mining associated soils are yet underexplored. It also points to the large proportion of the global diversity of bacteria yet uncultivated. Indeed, metagenomics studies of diverse environments are helping to increase our knowledge of bacterial diversity and to optimise cultivation strategies for bacterial species, which have previously been uncultivated. In the context of bioprospection, the findings of this study point to these soil environments as being rich in underexplored bacterial diversity, including species with economic relevance and potentials for improving soil health.

Several of the dominant genera and differentially abundant genera identified in this study, including *Acidibacter*, *Acidothermus*, *Bacillus*, *Bradyrhizobium*, *Burkholderia-Caballeronia-Paraburkholderia*, *Candidatus Udaeobacter*, *Candidatus Xiphinematobacter*, *Conexibacter* and *Sphingomonas* have been observed in the bacterial diversity of post-coal mining soils^18,45,46^. Overall, species of these genera contribute to soil nutrient cycling, biocontrol of plant diseases, promote plant growth, and modulate plant response to abiotic stress^18,55,56^. Specifically, species of *Microvirga* and *Bradyrhizobium* are plant-growth-promoting rhizobacteria which contribute to nitrogen fixation in the rhizosphere^18,57^, while *Bacillus* species are well known for their plant-growth-promoting ability, biocontrol of plant pest and pathogens, and the modulation of plant-hormone expression and adaptation to abiotic stress^55,56^. Similarly, the genera *Sphingomonas* comprise species with diverse functions in the soil ecosystem, including the degradation of polycyclic aromatic compounds^55^. *Candidatus Udaeobacter*, which was observed in all soils at relatively high abundance, is an oligotroph that can thrive in nutrient-poor conditions, thus suggesting, its potential use as an indicator species for reflecting poor soil nutrient state.

Within the context of ecological relevance and potential application of differentially abundant species in soil monitoring, both reclamation soils and reference soils harbour species (based on available information on characterised species) which have potentials for biocontrol e.g. *Lysobacter*, *Micomonospora*, *Dactylosporangium*, *Actinoplanes*, *Pseudonorcadia*, *Haliangium* and *Streptomyces*^58–60^; Nutrient mineralization e.g. *Dyella*, *Pseudaminobacter*, *Labrys*, *Pedomicrobium*, *Gemmatimonas*, *Sinomonas*, *Terrabacter*, *Mucilaginibacter*, *Conexibacter*, *Bryobacter* and *Candidatus Koribacter*^61^; plant-growth-promotion e.g. *Mesorhizobium*, *Microvirga*, *Bradyrhizobium and Solirubrobacter* and *Norcadioides*^18,57^; supporting soil ecological food web by being primary producers e.g. *Rhodoplanes*^62^; and pathogenicity e.g. *Burkholderia*, *Pajaroellobacter* and *Crossiella*^63^. With respect to ecological restoration of mining-disturbed areas, some of the potential plant-growth-promoting bacterial species could be isolated and employed as inoculum (biofertilizers) during post-mining reclamation—a practice that is currently not implemented in post-mining reclamation practices within South Africa. Thus, as a recommendation towards best practices for mediating post-mining restoration success, the inclusion of a microbial consortium in the current local reclamation practice should be considered. Ideally, such microbial consortium should comprise beneficial (e.g. plant-growth promoters, primary producers and nutrient-mobilizers) bacterial species and may be obtained from undisturbed or virgin sites using conventional methods for cultivating indigenous microorganisms^64,65^. In addition, based on observations from previous studies on stockpile^3,6,27^, the facilitation of microbial species proliferation and diversity during storage of topsoil in stockpiles (heaps) as well as the minimisation of compaction during soil replacement may aid soil health and consequently overall restoration success during post-mining reclamation.

Additionally, based on the growth requirement or adaptation of some characterised species to given environmental conditions, some differentially abundant genera may be further explored for potential utilization as bioindicators for reflecting soil conditions, including salinity (e.g. *Altererythrobacter*, *Pirellula*, and *Haliangium*^60^), acidity (e.g. *Bryobacter*, *Acidothermus* and *Singulisphaera*^66^), iron availability (e.g. *Candidatus Koribacter*), low nutrient availability (e.g. *Candidatus Udaeobacter*) and moisture availability (e.g. *Gemmatimonas*). Summarily, both reclamation and reference these soils are rich in microbial diversity and may serve as a suitable source for the bioprospection of novel species as well as species with potential industrial importance such as the production of secondary metabolites (e.g. antibiotics by Streptomyces species).

Unlike bacterial genetic diversity, the predicted functional diversity was not significantly influenced by soil history. This observation suggests redundancy in the functional capabilities of soil bacterial communities and agrees with results obtained in the phenotypic-based assays and the microbial community of fens^67^. Such redundancy in microbial community functional capabilities is linked to the resilience of soil bacterial community to environmental constraints and to the role of soil microbial communities in ensuring resilience in the soil ecosystem function^11,13^. The significant differences observed for specific predicted functions suggest that some bacterial communities are functionally more capable of regulating specific soil processes than others. However, because the prediction of bacterial community functions in this study is only based on associations of phylotypes, further studies are needed to investigate the presence and differential expression of several ecologically relevant functional genes in these soil environments.

## Conclusion

Without taking into cognisance site-specific differences, both phenotypic assays (selected enzyme activities and community level physiological profiling) and high-throughput sequence analysis of bacterial communities suggest that the bacterial communities and functional capabilities increase with age of reclamation. The upward trend in these indices suggests post-mining species restoration over chronological age. The bioprospection for some potential ecologically relevant phylotypes and their inclusion as inoculum during post-mining reclamation may contribute to mediating ecological restoration. Furthermore, the influence of selected soil properties such as pH and bulk density on bacterial communities suggest that post-mining reclamation practices must ensure minimising soil compaction, preserving pre-mining soil horizon and quality as well as include measures for soil pH amelioration.

Within the context of soil health monitoring, the structural differentiation in bacterial communities between reclamation and unmined areas, as well as between sites show that bacterial communities are sensitive to soil management practices and thus may reflect possible site-specific discrepancies in reclamation practices and soil properties. Hence, differentiation in the structure and function of bacterial communities is a potentially useful bioindicator which can be included as part of a minimum dataset for monitoring soil health and ascertaining the adequacy of current post-mining reclamation protocols towards soil ecosystem restoration.

## Methods

### Study site description and design

The study sites are all within the Grassland Biome of South Africa^68^. The altitude of the study area is 1400–1600 m above sea level. The annual maximum and minimum temperature for the study area is 31 °C and 10 °C, respectively, while total annual rainfall is 938 mm^69^. During the sampling period (April-May 2016), monthly average maximum and minimum temperatures were 24 ± 3 °C and 15 ± 3 °C, respectively, while average precipitation was 12.5 ± 10.27 mm and humidity 54 ± 5%^69^. Although the Highveld of Mpumalanga is the largest coal-mining region of South Africa, agriculture remains a key component of the province since the soils are highly arable^70,71^. Post-mining land reclamation is therefore paramount for the restoration of pre-disturbance conditions or other acceptable high-end land-use capabilities such as pasture lands for animal grazing. The procedure for reclamation in all three sites was according to the local guidelines. Briefly, different soil horizons—overburden, subsoil and topsoil (each stockpiled separately pre-mining)—are replaced in the same order, soil amelioration and fertilisation are applied before seeding with a mixed vegetation species (commonly *Eragrostis tef*, *Eragrostis curvula*, *Digitaria eriantha*, *Cynodon dactylon* and *Chloris gayana*)^21^. Herein, reclamation refers to land prepared and revegetated as described above. In addition, the ages of the reclaimed lands are the period between revegetation and time of sampling. As at the time of sampling, the unmined areas were not utilised for any anthropogenic activity (no evidence to suggest so). However, because the unmined areas are unprotected and close to urban amenities, these unmined areas can be impacted by activities such as road constructions and grazing.

The mean vegetation aerial cover of the reclamation sites was estimated (using 1 m^2^ quadrant) to be 30%, 35%, and 50% in ReclX, ReclY and ReclZ, respectively, with grass species (mostly *Eragrostis tef*) the dominant vegetation type across all sites. Bare patches of hard-crusted soils were however observed in reclamation soils, especially in ReclY. All reference sites were dominated by *Eragrostis tef* and had a vegetation cover >60%. Although the unmined sites are not impacted by mining, other anthropogenic activities such as road construction and grazing may have occurred.

### Sampling

Soil sampling was conducted in the autumn (April-May) of 2016. In each site, bulk soil samples were collected from the 0–15 cm depth using a sterile auger. Based on-site dimensions and for obtaining a representative sample, bulk soil was sampled from sites either along (at 10 m intervals) three parallel 40-m transects placed 100 m apart or from five points of five systematically positioned (100 m apart) crosses (see Supplementary Fig. S2). The three transects or five crosses served as replicates for each site. For both sampling designs, soil cores (1 kg each) collected along each transect or at points of each cross were pooled to form a composite sample.

For respective downstream analyses (DNA analysis, enzyme assays, community-level physiological profiling and physicochemical analyses), composite samples were aseptically divided into portions. Sample portions for DNA-based bacterial community analysis were frozen at −70 °C while portions for other analyses were passed through a 2-mm sieve and immediately stored: frozen for enzyme assays and physiological profiling as well as stored at room temperature for physicochemical analyses. Samples were analysed within a week of collection.

### Selected soil physicochemical analyses

The physicochemical properties of soil, including pH, texture (sand, silt and clay fractions), moisture content, organic matter (OM), bulk density (BD), electrical conductivity (EC), extractable cations (calcium, sodium, magnesium, potassium), anions (Cl^−^, NO_2_^−^, NO_3_^−^, PO_4_^3−^) and cation exchange capacity (CEC) were determined using standard procedures^72^ as described in the supplementary information (Text S1).

### Community-level physiological profiling (CLPP) of soil microbial communities

Carbon substrates utilization pattern in a 96-well Biolog EcoPlate (Biolog Inc., Hayward, CA, USA) was used to determine microbial community richness and evenness in soil^73,74^. For CLPP, 10 g of soil sample was suspended in 90 ml of sterile distilled water and shaken on a rotary shaker at 250 rpm for 1 h. The supernatant was further diluted (1:100 in sterile H_2_O), inoculated (150 µl) into the wells and incubated at 28 °C for 7 days, during which optical density (at wavelength 590 nm) measurements were taken twice daily to determine the average colour development within each well^73^. Optical density values were normalised prior to computing Shannon-Wiener index of diversity (*H*′) and evenness index (*J*′) based on the different number of substrates utilised and relative intensity as described by Habig *et al*.^73^.

### Determination of soil enzyme activities

Enzyme assays, β-glucosidase, acid- and alkaline- phosphatase and urease, were used to estimate functional activities of soil microbial communities in the mineralisation of carbon, phosphorus and nitrogen, respectively^34,36^. β-glucosidase, acid- and alkaline- phosphatases) were determined as described by Dick *et al*.^33^ while urease activity was determined according to Kandeler and Gerber^75^. Soil enzymatic activities were computed from extrapolations made from the standard curves of appropriate references^33,75^.

### 16S rRNA gene library preparation

The partial 16S rRNA gene (hypervariable V3-V4 region) was used as a barcode for characterising bacterial communities. Community DNA was extracted from 0.25 g of soil sample using the Power Soil DNA extraction kit (Qiagen, Hilden, Germany) according to the manufacturer’s protocol. The concentration of extracted DNA was quantified using a Qubit fluorometer (Invitrogen, Carlsbad, CA, USA) and normalised to equimolar concentrations (5 ng/µl) using 0.1 M Tris-HCl (pH 8.5) prior to PCR amplification with Illumina-barcoded 341 F and 805 R primers^76^. 16S rRNA library preparation was performed as described previously^24^. Paired-end (2 × 300 bp) sequencing of the partial 16S rRNA gene libraries was performed on the Illumina MiSeq sequencer using the Nextera v3 kit (Illumina Inc., San Diego, CA, USA) at the Agricultural Research Council-Biotechnology Platform, Pretoria, South Africa.

### Bioinformatics

Sequence reads were demultiplexed and trimmed of barcodes using the MiSeq Reporter software (Illumina Inc, San Diego, CA, USA). Demultiplexed reads were quality checked using FastQC (v. 0.11.5, Babraham Bioinformatics, UK), quality trimmed using Trimmomatic software (v. 0.36)^77^ and assembled using PANDASeq software (v. 2.10)^78^ as described in the supplementary information (Text S2). Operational taxonomic units (OTUs) were picked at 97% 16S rRNA gene similarity in QIIME software (v. 1.9.1)^79^ by using both “open reference picking” (for taxonomic diversity analyses) and “closed reference picking” (for predicted functional profiling) strategies against the SILVA rRNA database (release 132)^80^ with usearch61 reference^81,82^ to eliminate chimeras. The OTU count tables (for closed and open reference OTU picking strategies) were depleted of singletons and non-bacterial phylotypes (e.g. archaea and unassigned to domains) before single rarefaction (normalisation) to an even depth of 19 500 sequences per sample. Thereafter, alpha and beta diversity analyses were performed in QIIME software and/or R software (v. 3.5.3)^83^.

### Predicted functional metagenomic profile of bacterial communities

The functional metabolic profile (enzyme-coding genes) of soil bacterial community was predicted by using the Tax4Fun package^25^ of R software. Tax4Fun transforms the SILVA-based OTU count table into functional or metabolic profiles by using normalised 16S rRNA copy numbers and a set of pre-computed metabolic reference profiles based on the Kyoto Encyclopaedia of Genes and Genomes (KEGG) database (http://www.genome.jp/kegg/)^25,84^. The functional prediction was performed in terms of the KEGG Orthology (KO) terms. In order to investigate if specific soil bacterial community functions are influenced by site and soil history, only a subset of the KO terms involved in the metabolism of key soil nutrients such as carbon, phosphorus and nitrogen, were statistically analysed as described in the next section.

### Statistical analyses

Except stated otherwise, all statistical analyses were performed in R software (v. 3.5.3)^83^. Data (physicochemical properties, and physiological-based) was transformed to fit a normal distribution by either log_10_, square-root, sine transformations. Where normality could not be achieved through transformation, non-parametric tests were used on the raw data. Statistical analyses of physiological and bacterial diversity data were performed under a nested mixed effect model. For normally distributed data, a linear mixed model with restricted maximum likelihood estimation method was performed in the “lm4” package of R software^85^. Whereas, for non-normally distributed data, a generalised linear mixed model with a Gaussian log-link function and a penalised quasi-likelihood estimation method was performed using the “glmmPQL ()” function in the “MASS” package of R software. In the mixed models, “site” and “history” (reclamation vs. unmined) were set as fixed factors while age (coded as a categorical variable in which same values were assigned to unmined soils and different values assigned for each reclamation site) was set as a random variable nested within “history”. Model assumptions were confirmed by inspecting residual plots, while pair-wise posthoc tests were performed by using the “Tukey” adjustment in the “emmeans” package (v. 1.3.5.1) of R software. The relationship between soil physicochemical parameters and physiological data (enzymes and CLPP—based microbial diversity indices) was tested by performing either a Pearson correlation on pairs of normalised data or Spearman rank correlation where data could not be transformed to near-normality.

For multivariate analyses of microbial community data (97% 16S rRNA gene similarity OTUs), relative proportion of each OTU count within a sample data were log-transformed (log (x) + 1, where x > 0)^86^ by using the “decostand ()” function in the vegan package (v.2.55)^87^ of R software. Visualisation of community structure in multivariate space was performed using a non-metric dimensional scaling and an unweighted pair-group method with arithmetic mean (UPGMA) by using the vegan and dendextend (v. 1.12.0)^88^ packages of R software, respectively. Test for differences in multivariate space was performed on the Bray-Curtis dissimilarity using permutational multivariate analysis of variance (PERMANOVA) and Permutational test for homogeneity of multivariate dispersions (PERMDISP). The Linear Discriminant Analysis (LDA) Effect size (LEfSe)^89^ was further performed to detect differentially abundant phylotypes (Mann-Whitney U test, *P* < 0.05, LDA score > 2) between sample groups and further visualized in an annotated cladogram using the GraPhlAn software (v. 0.9.7)^90^ or as bar plots using the web-based Microbiome Analyst tool (www.microbiomeanalyst.ca)^91^. For predicted KEGG orthology (KO) terms, an indicator species analysis^92^ was additionally performed to detect KO terms which most discriminatory between pairs of reclamation and reference soils at each site (see supplementary Text S3). In this study, KO terms with FDR-adjusted *P* < 0.1 and an indicator value > 0.6 was adjudged discriminant between reference and reclamation soil.

Lastly, in order to elucidate which soil physicochemical parameters best explain variations in microbial community composition across sites, a canonical correspondence analysis (CCA) was performed using an automatic stepwise model selection (“ordistep ()”) in the vegan package. Variables with multicollinearity (variance inflation factor > 10) were excluded from the final plot. For CCA, log-transformed physicochemical data and bacterial species composition relative counts (>1% at the genus-level) were used. Significance for all tests was set at probability (*P*) < 0.05.

## Supplementary Information

Supplementary Information.

## Data Availability

16S rRNA sequence data generated in this study are available in the sequence read archives (SRA) of the National Centre for Biotechnological Information as part of a BioProject under the SRA accession number PRJNA526293 (https://www.ncbi.nlm.nih.gov/bioproject/PRJNA526293/).
