# Supplementary material for: Structural and functional differentiation of bacterial communities in post-coal mining reclamation soils of South Africa: bioindicators of soil ecosystem restoration
Source: Sci Rep. 2020 Feb 4;10:1759. doi: 10.1038/s41598-020-58576-5 (PMC7000389; doi:10.1038/s41598-020-58576-5)
Supplement: Supplementary file 1 — Supplementary Information. [file 41598_2020_58576_MOESM1_ESM.pdf]

Supplementary Information

Title: Structural and functional differentiation of bacterial communities in post-coal mining reclamation soils of South Africa: bioindicators of soil ecosystem restoration

Authors: Obinna T. Ezeokoli[1,2,3], Cornelius C. Bezuidenhout[1], Mark S. Maboeta[1], Damase Khasa[3,4], Rasheed A. Adeleke[1,2*]

Affiliations

[1]Unit for Environmental Sciences and Management, North-West University, Potchefstroom 2520, South Africa.

[2]Agricultural Research Council-Institute for Soil, Climate and Water, Arcadia, 0001, Pretoria, South Africa

[3]Institute for Integrative and Systems Biology, Universite Laval, Quebec City, QC G1V 0A6, Canada

[4]Centre for Forest Research, Faculty of Forestry, Geography and Geomatics, Universite Laval, Quebec City, QC G1V 0A6, Canada

*Correspondence: Rasheed.Adeleke@nwu.ac.za.

Supplementary Text (Methods)

Text S1: Brief method description of physicochemical analyses

Briefly, pH was determined from a 1:2.5 soil-water suspension using a pre-calibrated pH meter (pH 700, Eutech Instruments Pte Ltd, Singapore). Particle size distribution was determined by the Bouyoucos method[1]. Cations and exchangeable cations were determined from ammonium acetate (1 M, pH 7) soil extracts using Inductively coupled plasma - optical emission spectrometry. Anions were determined from water extracts using Ion-exchange chromatography. BD determination was done after overnight drying (at 105°C) of soils collected on-site with a BD sampler.

Text S2: Bioinformatics Analyses-quality trimming

Quality trimming was performed using Trimmomatic software as follows: poor quality trailing and leading nucleotide positions were first trimmed from both forward and reverse reads. Thereafter, reads with an average quality score (Phred, Q) less than 20 and read length less than 250 bp were eliminated. For assembly, quality-trimmed forward and reverse reads were assembled and filtered for ambiguous bases ("N") and spurious length (assembled read length 420 bp $\geq$ L $\leq$ 466 bp) by using the Simple Bayesian algorithm and a threshold of 0.7 in PANDASeq software (v. 2.10)[2].

Text S3: Indicator species analysis

Indicator species analysis assigns an indicator value (between 0 and 1) to each species in the cluster or group based on the product of the relative abundance and relative frequency of that species within the cluster (reclamation or reference). A high indicator value (close to 1)

suggests that a given species is highly abundant within a group compared to the other group (or groups) (referred to as “specificity”) and is present in most members of that group (referred to as “fidelity”)[3]. Tests for statistical significance of the indicator value was further determined through permutation (probability) tests[4]. In this study, the indicator species analysis was performed using the “indval ()” function in the labdsv package of R software [4]

In this study, KO terms with FDR-adjusted $P < 0.1$ between reclamation and reference soils of at least one site and with an indicator value > 0.6 was adjudged discriminant between reference and reclamation soil.

Supplementary Tables

Table S1. Selected soil physicochemical properties

	Site X		Site Y		Site Z	
Properties[1]	Recl.	Ref.	Recl	Ref.	Recl	Ref.
Cl^- (mg kg^{-1})	1.05±0.28[a]	1.42±0.94[a]	0.94±0.32[a]	1.06±0.07[a]	1.04±0.49[a]	0.88±0.72[a]
Na (mg kg^{-1})	3.22±0.40[a]	4.05±1.42[a]	15.01±10.42[a]	6.67±1.84[a]	2.07±0.88[a]	7.04±8.77[a]
K (mg kg^{-1})	22.88±1.58[a]	40.56±23.35[a]	79.27±13.28[a]	75.27±14.43[a]	77.30±17.09[a]	69.50±19.47[a]
Ca (mg kg^{-1})	42.80±10.82[b]	176.48±126.09[a]	322.85±74.22[a]	1371.90±7.50[b]	237.08±45.47[a]	226.84±120.26[a]
Mg (mg kg^{-1})	10.76±2.22[b]	37.01±20.27[a]	147.03±35.52[b]	358.32±138.24[a]	57.89±32.05[a]	43.39±22.58[a]
Textural class	SaClLm	SaLm	SaClLm	SaLm	SaClLm	SaLm

[1]See also Table 1 for other physicochemical properties.

Table S2: Community-level physiological profiling (31 Carbon substrate utilization pattern)

Site	Soil group (Sample size)	Shannon-Weiner (*H*')	Evenness (*J*´)
Site X	ReclX (N=3)	1.81±1.08	0.62±0.33
	RefX (N=5)	1.86±0.51	0.79±0.08
Site Y	ReclY (N=5)	1.68±0.58	0.73±0.21
	RefY (N=3)	1.91±0.20	0.69±0.07
Site Z	ReclZ (N=5)	1.37±0.89	0.61±0.30
	RefZ (N=5)	1.36±0.72	0.60±0.28

Values (mean ± SD). Differences are not significant ($P < 0.05$) based on a mixed effect model (Random effect, variance = 0.001145, Standard deviation = 0.03384).

Table S3: Permutational tests for microbial community structure between reclamation and reference soils per site based on Bray-Curtis distances

Factors	Unweighted Bray (composition)			Weighted Bray (Structure)		
	PERMANOVA		PERMDISP	PERMANOVA		PERMDISP
	R^2 (%)	*P*	*P*	R^2 (%)	*P*	*P*
Pair-wise site comparison						
Site X	29.20	0.018	0.001	26.95	0.090	0.001
Site Y	53.09	0.018	0.120	51.50	0.026	0.119
Site Z	13.58	0.280	0.035	12.66	0.406	0.058
Sample-wide analyses						
Site	27.29	0.001	0.22	28.16	0.001	0.263
Soil History	6.70	0.007	0.04	8.13	0.004	0.032
Site x Soil History	13.65	0.001	ND	14.80	0.001	ND

ND, Not determined. PERMANOVA tests were performed by using the “adonis ()” of the vegan package of R software and are based on 999 iterations.

Table S4: Statistical test for discriminative genus-level features between reclamation and reference soil. See Figure 4.

	P values	FDR- adjusted P-values	Class	LDA score
Massilia	0.003	0.337	Reclamation	4.65
Sporosarcina	0.004	0.337	Reclamation	3.49
Oryzihumus	0.004	0.337	Reclamation	4.36
Terrabacter	0.005	0.337	Reclamation	4.09
Mucilaginibacter	0.006	0.337	Reclamation	3.72
Oceanobacillus	0.007	0.337	Reclamation	2.66
Janibacter	0.007	0.337	Reclamation	3.97
Sphingomonas	0.008	0.337	Reclamation	5.2
Deinococcus	0.009	0.337	Reclamation	2.74
Rhodanobacter	0.012	0.337	Reclamation	3.61
Dokdonella	0.013	0.337	Reclamation	3.43
Segetibacter	0.014	0.337	Reclamation	3.9
Phycicoccus	0.014	0.337	Reclamation	3.73
Dyella	0.016	0.337	Reclamation	3.73
Fulvimonas	0.017	0.337	Reclamation	3.06
Streptomyces	0.019	0.337	Reclamation	4.41
Clostridium sensu stricto *1*	0.021	0.337	Reclamation	3.08
Opitutus	0.026	0.380	Reclamation	3.42
Arthrobacter	0.026	0.380	Reclamation	3.82
Flavisolibacter	0.026	0.380	Reclamation	4
Methylobacterium	0.034	0.383	Reclamation	3.63
Candidatus *Koribacter*	0.034	0.383	Reclamation	3.8
Jatrophihabitans	0.034	0.383	Reclamation	4.04
Clostridium sensu stricto 12	0.040	0.383	Reclamation	2.75
Burkholderia-Caballeronia-Paraburkholderia	0.005	0.337	Reclamation	4.43
Rubrobacter	0.003	0.337	Reference	-3.6
Vicinamibacter	0.007	0.337	Reference	-3.49
Chitinophaga	0.014	0.337	Reference	-2.97
Lechevalieria	0.018	0.337	Reference	-3.59
Chryseolinea	0.020	0.337	Reference	-2.08
Sphingomicrobium	0.020	0.337	Reference	-2.09
Rhodopirellula	0.020	0.337	Reference	-2.53
Herpetosiphon	0.020	0.337	Reference	-2.56
Hirschia	0.020	0.337	Reference	-2.8
Flavitalea	0.029	0.383	Reference	-3.05
FFCH5858	0.030	0.383	Reference	-2.64
Virgisporangium	0.030	0.383	Reference	-2.67
SWB02	0.032	0.383	Reference	-2.83
Candidatus *Protochlamydia*	0.037	0.383	Reference	-2.54

Table S5: Tax4Fun statistics for the functional prediction of soil bacterial communities

Soil group	Average FTUs	†1-Average FTUs
ReclX	0.89±0.02[a]	0.11±0.02[a]
RefX	0.86±0.03[a]	0.14±0.03[a]
ReclY	0.81±0.04[a]	0.19±0.04[a]
RefY	0.82±0.01[a]	0.18±0.01[a]
ReclZ	0.84±0.01[a]	0.16±0.01[a]
RefZ	0.85±0.04[a]	0.15±0.04[a]

FTU, Fraction of OTUs which were not mapped against KEGG organisms.

†Fraction of OTUs which mapped onto the KEGG organisms is obtained by subtracting FTUs from 1.

Table S6: Pearson correlation coefficient (r) for the relationship between soil physicochemical properties and soil physiological data

Physico-chemical ppts.	Beta-glucosidase (P-nitrophenol µg/g/h)	Alk-phosphatase (P-nitrophenol µg/g/h)	Acid-phosphatase (P-nitrophenol µg/g/h)	Urease (NH_4-N µg/g/2h)	Shannon-Weiner (*H´*)	Evenness (*J´*)
	r	r	r	r	r	r
pH (H_2O)	0.40	0.653**	0.103	0.64**	0.223	0.003
Moisture (%)	0.439*	0.374	0.275	0.443*	0.164	0.046
Organic matter (%)	0.064	0.188	-0.024	0.253	0.091	-0.097
Bulk Density (g cm^{-3})	-0.335	-0.133	-0.375	-0.265	-0.100	-0.239
EC (mg kg^{-1})	-0.299	-0.442*	-0.337	-0.256	-0.222	-0.0.299
Cl^- (mg kg^{-1})	-0.089	-0.319	-0.265	-0.178	-0.397	-0.090
NO_3^--N (mg kg^{-1})	0.015	0.178	-0.084	-0.299	0.248	0.020
NO_2^- -N (mg kg^{-1})	0.334	0.418	-0.080	0.346	0.483*	0.195
PO_4^{3-} -P (mg kg^{-1})	-0.254	-0.201	-0.059	-0.433	-0.240	-0.167
Na (mg kg^{-1})	-0.327	0.080	-0.532**	-0.052	0.194	0.020
K (mg kg^{-1})	0.460*	0.207	0.417*	0.602**	-0.100	-0.188
Ca (mg kg^{-1})	0.561**	0.510**	0.065	0.678**	0.144	-0.126
Mg (mg kg^{-1})	0.351	0.478*	0.063	0.508*	0.093	-0.091
CEC (cmol (+) kg^{-1})	0.162	0.273	-0.355	0.100	0.286	0.055
Sand (%)	-0.08	-0.179	0.15	-0.13	-0.25	-0.239
Silt (%)	0.34	0.1.69	0.169	0.32	0.02	0.042
Clay (%)	-0.428*	-0.369	-0.27	-0.258	-0.452*	-0.332

*Correlation is significant at 0.05 probability level.

** Correlation is significant at the 0.01 probability level.

Table S7: Significance of terms (physicochemical properties) in the CCA model of Fig. 9.

Constraints	F	Pr. (>F)
Sand	2.2023	0.136
Silt	5.5790	0.013*
Clay	1.6856	0.199
BD	4.3089	0.022*
pH	25.7254	0.001***
Moist	1.0265	0.360
OM	1.8720	0.164
EC	5.4034	0.015*
Cl	1.5691	0.221
NO_3	2.0760	0.174
NO_2	1.4687	0.260
PO4	1.3124	0.272
Na	3.4884	0.041*
K	0.9829	0.393
Ca	4.5304	0.026*
Mg	1.0584	0.376
CEC	1.5399	0.222

* Significant at the 0.05 probability level

**Significant at the 0.01 probability level

***Significant at the 0.001 probability level

Supplementary Figure

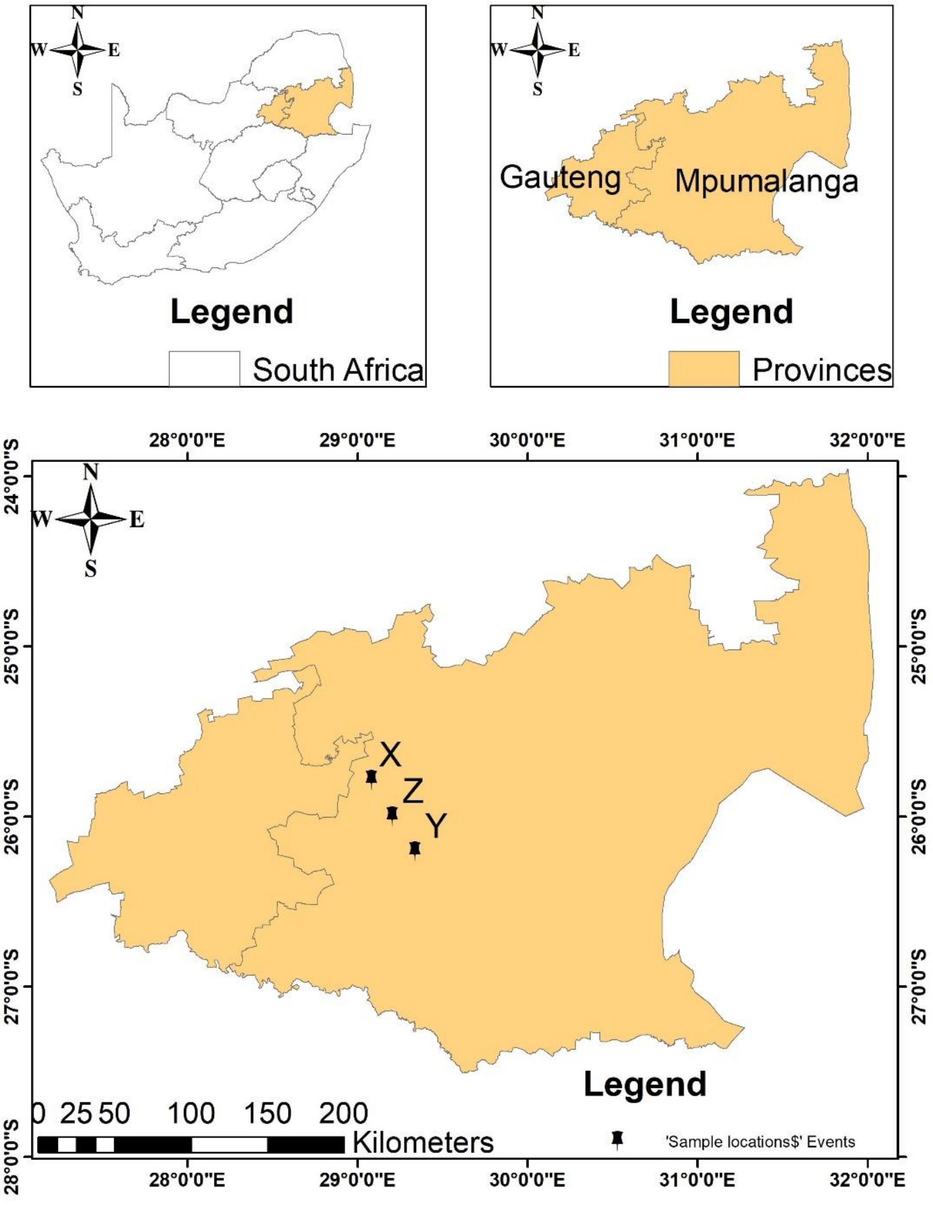

Figure. S1

Figure S1: Map of the sampling area. Site Y is approximately 67 km to the south of site X; site Z is approximately 32 km to the south of site X, while site Z is approximately 41 km to the north of site Y. Map was generated using the ArcMap software (v. 10.5; Esri, Redlands, CA, USA) using a shapefile obtained from the North-West University University's (www.nwu.ac.za) local Geo-Database archives.

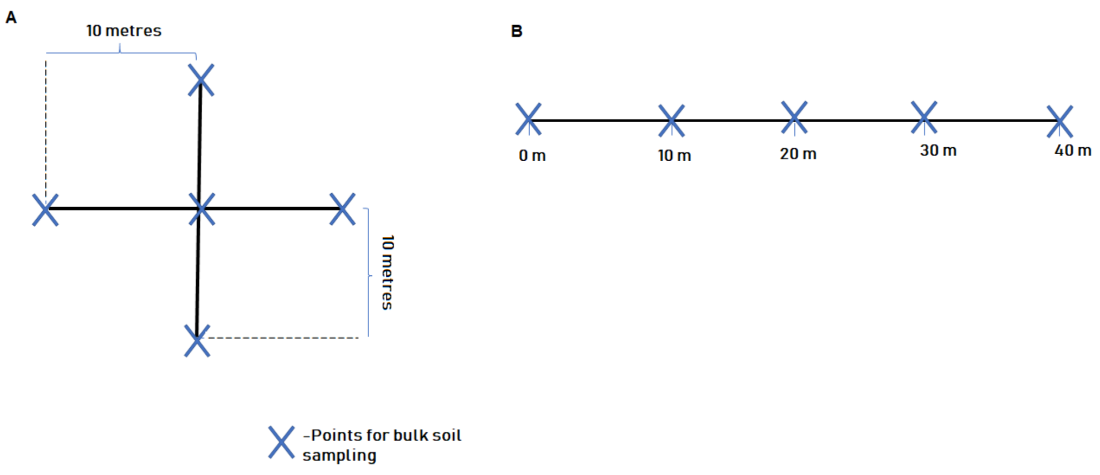

Figure S2

Figure S2. Schematic diagram for soil sampling designs. (A) Cross design. (B) Transect method. The sampling design was aimed at obtaining representative samples and differed based on the topography and dimension of the sites. Transect method was used in site X, while cross designs were applied to site Y and Z. Samples were collected from each sampled area using 3-5 transects or crosses which served as replicates.

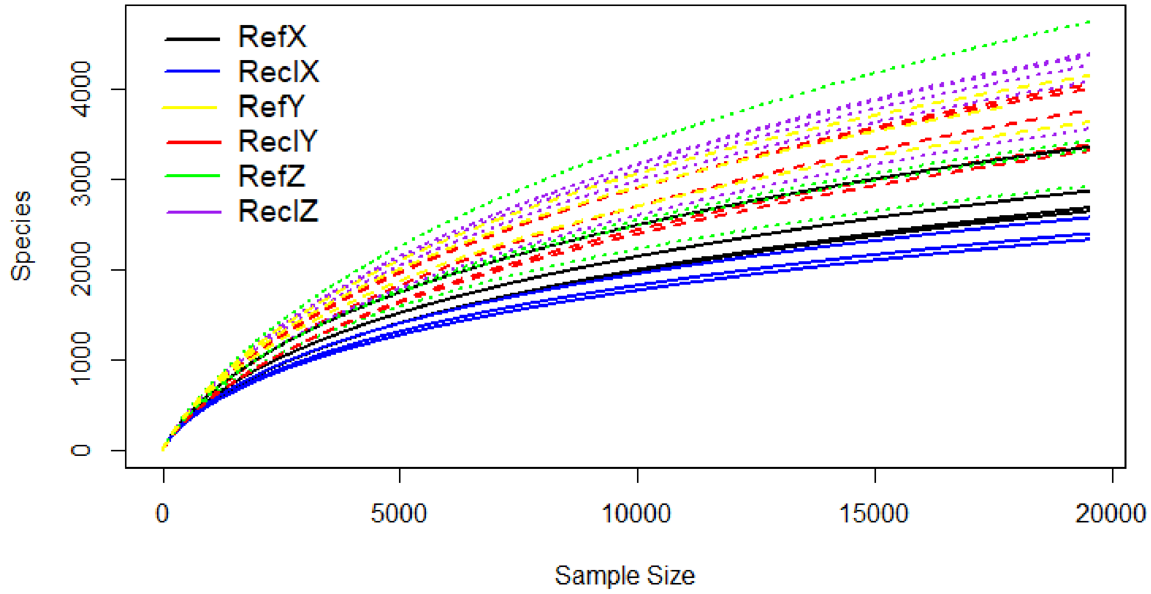

Figure S3. Rarefaction curve**.** Bacterial communities (97% 16S RNA gene similarity OTUs) were subsampled at a depth of 19500 sequences per sample. The absence of a plateau in most sites suggest that the richness of the community is underrepresented.

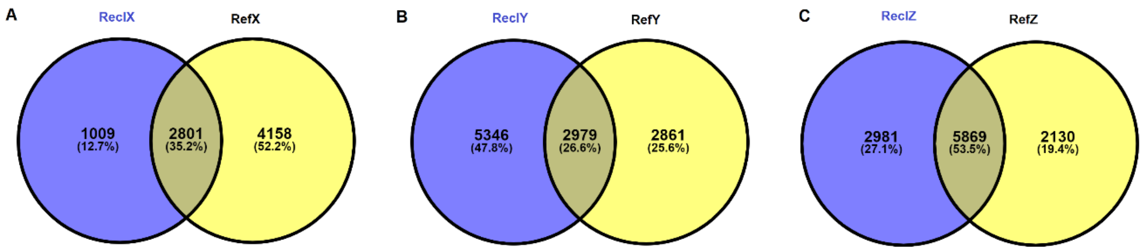

Figure S4

Figure S4. Unique and shared OTUs between and within sites. (A) Site X. (B) Site Y. (C) Site Z. Total number of OTUs per set (or soil cluster) is the sum of unique OTUs in all replicates for each soil group (e.g. site or history). The proportion (expressed in percentage) of OTUs within each subset with respect to the total number of OTUs for any given sets are provided in parenthesis. Venn diagram was constructed by using the online Venny 2.1 software available from http://bioinfogp.cnb.csic.es/tools/venny/.[5]

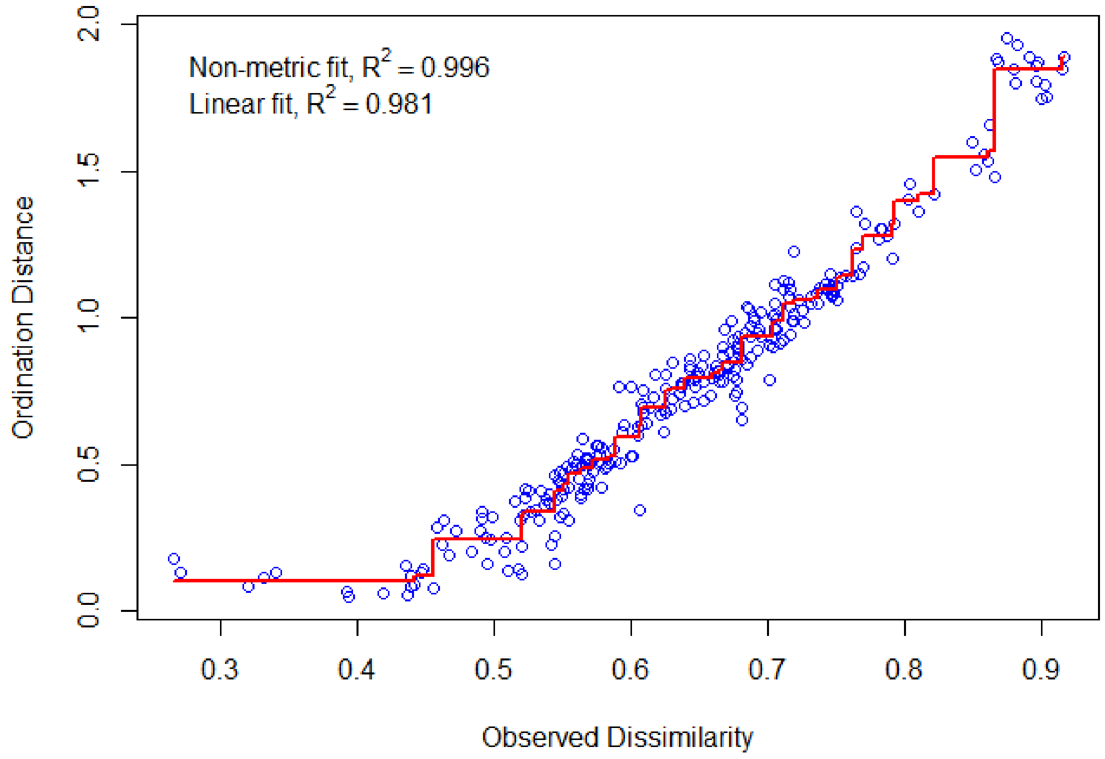

Figure S5

Figure S5. Stress plot for the non-metric multidimensional scaling plot of Figure 2a. Stress plot was generated using the "stressplot ()" function of the Vegan package of R software[6,7].

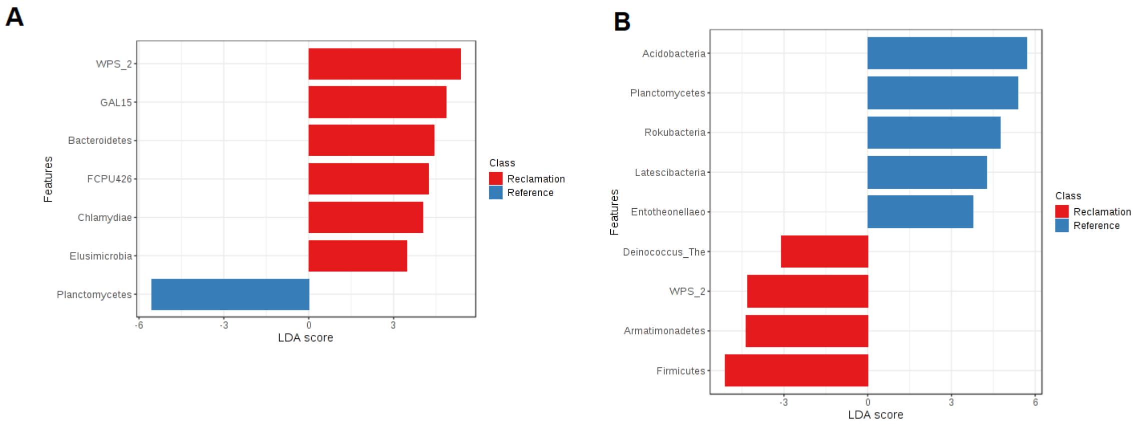

Figure S6

Figure S6. Differentially abundant phyla between bacterial communities of reclamation and reference soils (A) Differentially abundant phyla (LDA score > 2.0, FDR-adjusted *P*-value < 0.1) in site X. (B) Differentially abundant phyla (LDA score > 2.0, FDR-adjusted *P*-value < 0.3) in Site Y. Differential abundance and bar plots were determined and generated, respectively, using LefSe[8] via the Microbiome Analyst (www.microbiomeanalyst.ca)[9].

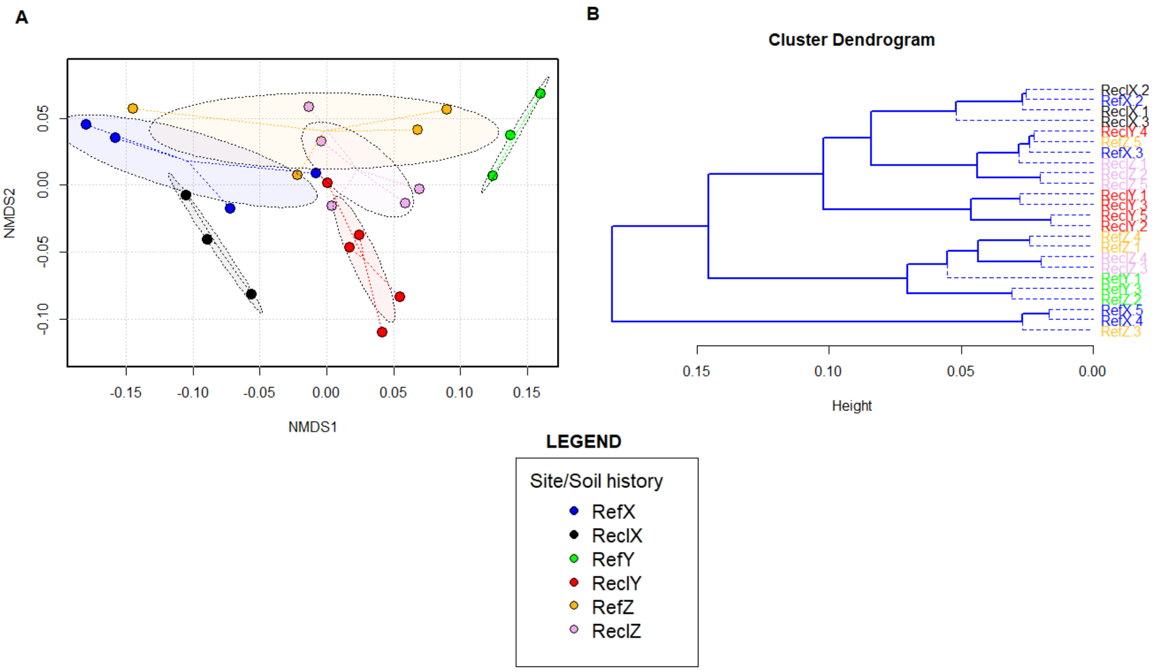

Figure S7

Figure S7. Bray-Curtis dissimilarity for predicted functional profile of soil bacterial communities. (A). Non-metric dimensional scaling plot. (B). UPGMA hierarchical cluster dendrogram. Dotted lines in the nMDS plot show the distance of every sample to its group centroids in multivariate space, while ellipses show 95% confidence intervals (standard error) in multivariate space around group centroids. The stress of the nMDS plot is 0.03. Differences in multivariate space are significant for site and history interactions (PERMANOVA R^2 = 7.91%, P = 0.045; PERMDISP P = 0.016).

References

1 Bouyoucos, G. J. Hydrometer method improved for making particle size analyses of soils 1. *Agron. J.* **54**, 464-465 (1962).

2 Masella, A. P., Bartram, A. K., Truszkowski, J. M., Brown, D. G. & Neufeld, J. D. PANDAseq: paired-end assembler for illumina sequences. *BMC Bioinformatics* **13**, 31, doi:10.1186/1471-2105-13-31 (2012).

3 Dufrene, M. & Legendre, P. Species assemblages and indicator species: the need for a flexible asymmetrical approach. *Ecol. Monogr.* **67**, 345-366 (1997).

4 Roberts, D. W. labdsv: Ordination and multivariate analysis for ecology. *R package version* **1** (2007).

5 Oliveros, J. C. *Venny. An interactive tool for comparing lists with Venn's diagrams* http://bioinfogp.cnb.csic.es/tools/venny/index.html (2007-2015).

6 Oksanen, J. *et al. vegan: Community Ecology Package* https://CRAN.R-project.org/package=vegan. (2019).

7 R: A language and environment for statistical computing (R Foundation for Statistical Computing, Vienna, Austria, 2013).

8 Segata, N. *et al.* Metagenomic biomarker discovery and explanation. *Genome Biol.* **12**, R60 (2011).

9 Dhariwal, A. *et al.* MicrobiomeAnalyst: a web-based tool for comprehensive statistical, visual and meta-analysis of microbiome data. *Nucleic Acids Res.* **45**, W180-W188 (2017).
